# The trajectory of crack users to the street situation in the perspective of family members

**DOI:** 10.17533/udea.iee.v37n2e03

**Published:** 2019-09-19

**Authors:** Maycon Rogério Seleghim, Sueli Aparecida Frari Galera

**Affiliations:** 1 Nurse, Post-Doctor, Professor at the Ribeirão Preto College of Nursing of University of São Paulo, Brazil. Email: mseleghim@yahoo.com.br Universidade de São Paulo University of São Paulo Brazil mseleghim@yahoo.com.br; 2 Nurse, Post-Doctor, Associate Professor at the Ribeirão Preto College of Nursing of University of São Paulo, Brazil. Email: suugalera@eerp.usp.br Universidade de São Paulo University of São Paulo Brazil suugalera@eerp.usp.br

**Keywords:** crack cocaine, family relations, homeless persons, community mental health services., cocaína crack, relaciones familiares, personas sin hogar, servicios comunitarios de salud mental.

## Abstract

**Objective.:**

To understand the family experience regarding the trajectory of crack users for the street situation.

**Method.:**

Qualitative study using the systemic approach as the theoretical referential and the narrative as methodological referential. We conducted interviews with eleven family members of crack users with street situation experience cared for at a community mental health service. We analyzed the interviews using the inductive content analysis technique.

**Results.:**

The family members understood the trajectory of the crack users for the street situation from two perspectives. One before the street situation process, for which they described a problematic childhood, the presence of stressor traumas/events, vulnerabilities in the family environment, and their family members' encounter with the drug world. Moreover, another posterior to the street situation, for which they narrated the perception of alterations in the users, the discovery of crack use, the deepening of the individuals' relationship with the streets, and the adoption of coping strategies.

**Conclusion.:**

It was made evident that the family adopts an explicative model for the behavior of drug use and contact with the streets based on the life history of the crack user family member.

## Resumen

**Objetivo.:**

Comprender la trayectoria de usuarios de crack en situación de calle desde la perspectiva de los familiares.

**Métodos.:**

Estudio cualitativo, realizado en un servicio público de salud mental, que utiliza el abordaje sistémico como referencial teórico y la narrativa como referencial metodológico. Se realizó una entrevista en profundidad con cuestión orientadora junto a 11 familiares, y la técnica de análisis de contenido para la exploración de las narrativas.

**Resultados.:**

Los familiares comprendieron la trayectoria para la situación de calle a partir de dos perspectivas principales: el pasado, donde narraron eventos de la infancia / adolescencia de los usuarios, y el presente, donde contaron específicamente sobre la ocurrencia de la situación de calle.

**Discusión.:**

Los eventos que justifican el uso de drogas son percibidos como momentos de fragilización, a partir del entendimiento de que el usuario era un "niño-problema".

**Conclusión.:**

Se evidenció que la creencia familiar puede funcionar como los ajustes para cuidar y mantener la unión y la homeostasis familiar.

## Introduction

The phenomenon of crack usage has elicited the attention of the government and the Brazilian and international society due to the social, health, and family problems experienced by many of its users. The experience of these problems may get to the extreme of favoring the occurrence of street situation experiences,(1) thus intensifying the susceptibility to the involvement with violent and illicit activities, compulsive use pattern of the drug, increased risk of contamination by agents that cause transmissible diseases such as hepatitis, tuberculosis, and HIV, marginalization, and difficulties to remain with the families.(2,3) Although studies carried out in the last decades indicate that the street situation is caused by a multitude of factors that vary from the economic and structural dynamics of the society to personal and psychological reasons, crack users in Brazil as in other countries have been expanding the characteristics of ties with the streets.(4)

It is known that the street condition phenomenon in crack users does not occur instantly, but is part of a process of weakening of the bonds in the social and family environment.(5) In the literature, one may observe the presence of two primary chains of study on the familiar aspects involved in the trajectory towards the street situation in drug users. On one side, there are studies concerned with studying the familiar characteristics such as causal or predisposing factors for the occurrence of the street experience(5,6) while, on the other, are studies, still incipient, related to the role of the family in preventing and solving the street experience.(7)

The primary problem of the studies that investigate the family as the cause of the street situation is that they collect data from the reports of people that are living in the streets. Therefore, they present only the viewpoint of this particular group. Hence, the family, as the primary source of information, is disregarded. Regarding the understanding of the role of the family, one may observe that the majority of the studies adopt the paradigm of linear causality, in which the families are considered causers of the drug use and of the street situation for presenting environmental and/or relational characteristics or risk factors. Adopting a circular causality model(8), in which it is understood that the behavior of drug abuse impacts the family dynamics and that the family dynamics impacts the chemical dependency, allows removing the family from the causer role. Moreover, it widens the focus to the family relations and offers space to the experience of family members with an individual in the context of drug abuse and street situation. In this sense, the objective of the current study is to understand the family experience regarding the trajectory of crack users to the street situation.

## Methods

This qualitative study employed the systemic approach as the theoretical referential and the narrative as a methodological referential (9^)^ to interview family members of crack users with street situation experience. The adoption of a systemic theoretical body was initially guided by two primary aspects. First, by the understanding that the street situation is not a phenomenon with consequences only for one of the family members, but has consequences for the entire family group. Therefore, the systemic approach allowed investigating, albeit from an individual perspective, the relationships that exist within a wider family context, providing important aspects regarding the family as a unison and inseparable group. Second, by the need to change the current paradigm of approach to the family group: instead of looking within the families, especially in family relations, for the causes of the drug use and the street situation, we sought, in this study, to understand how they experienced, understood, and acted in the face of such events.

A narrative may be defined as a textual form that allows translating the knowing into telling, shaping the human experience into an assimilable form of meaning structures.(10) Its articulation point with the systemic approach is concentrated mainly on the fact that the narrative is not just the report of an individual experience, but is built through dialogues to described experiences shared by members of a family, group, or community.(11) Therefore, the narrative consists of a form of establishing the vision of the individuals in the world, insofar as they situate the events and actions into stories instituted in the temporal order of what was experienced.(12) Upon telling and interpreting experiences, the narrative establishes a mediation between the interior world of thought-feeling and an exterior world of observable actions and behaviors.(13)

Study location. The study participants were selected in a Psychosocial Care Center for Alcohol and Drugs (CAPS ad) of a city in the state of São Paulo, Brazil. Before starting data collection, the first author frequented the CAPS to get to know the dynamic of the service and be able to get close to possible participants. The researcher has experience in conducting interviews with family members.

Participants. Family members of crack users with experience in a street situation, assisted by the mentioned service, in the period from June 2014 to February 2015. The street situation experience was defined as moments in which the users left their homes and started living in the streets as a result of crack use. The inclusion criteria were being at least eighteen years old, having some degree of kinship with the user, and having followed their problem with crack use and with the occurrence of the street situation. The initial methodological design predicted selecting only the participants in the therapeutic family groups. However, due to the low number of subjects that met the inclusion criteria and considering the non-emergence of new cases in the third month of data collection, we resorted to consulting the service professionals. In total, we interviewed eleven family members of eleven crack users.

Data collection. The approach and invitation to family members of the three therapeutic groups were carried out by the primary researcher at the beginning of each group session. The family members interested in participating in the interview sought the researcher at the end of the session. Depleting the possibilities of finding family members in the groups, the researcher asked the professionals to indicate family members that met the research criteria. Such family members were approached through telephone contact using a single text elaborated for this purpose. The family members that accepted to participate in the study were scheduled to attend the service. Considering the criteria for quality in qualitative research,(14) all participants were interviewed twice in a period not exceeding fifteen days after the first interview. The objective of the second interview was to confirm the information provided in the first, as well as explore and deepen the issues that had not been made clear and were important for contemplating the studied phenomenon. All the interviews were carried out in a reserved and private room of the service itself, digitally recorded in full, and lasted around thirty to forty minutes. The following data collection instruments were used: 1) questionnaire with socioeconomic and demographic information of the family members and the families, 2) questionnaire with data on the users, and 3) in-depth interview with narrative and systemic focus. The following guiding question was used: "We know that when people use crack, they may leave their homes and go through some periods in the streets. In this sense, could you tell me how this happened with your family member? How did you cope with this?" The dataset received an alphanumeric code according to the sequence in which the interviews took place.

Data analysis. The two interviews were grouped and transcribed in full. For the exploration of the material, the inductive content analysis technique was used, which is constituted of three phases:(14) 1) preparation phase - identification of the meaning that was contained in a given statement and/or excerpt through repeated readings, seeking to learn "what was going on" starting from the whole; 2) organization phase - included the open encoding (typing of notes in the margins of the text), elaboration of encoding spreadsheets (gathering of typed notes in a data spreadsheet), clustering (summarization of the data through the clustering of the notes), categorization and abstraction (elaboration of subcategories and categories through the classification of data as "belonging" or "non-belonging" to a given group); and 3) report generation phase - description of the results obtained with the analysis. This process was carried out by two researchers, with the generated categories being validated by a team of four judges who were researchers in mental health. The categories were sent previously to each judge that should analyze if the narratives were represented in the category. The intensity of agreement among the judges was measured using the Kappa coefficient, whose value was of 1.00 - i.e., an almost perfect agreement level. Thus, by the end of the analysis, the data were organized into two main categories, each with four subcategories. 

The study was authorized by the service and approved by the research ethics committee with protocol CAAE 34923414.9.0000.5393. All ethical norms were respected.

## Results

Of the eleven family members interviewed, nine were mothers, with an average of 57 years old, evangelical (7), and coming from less privileged areas of the municipality where the research took place (9). Regarding the characteristics of the families, we verified that they were composed of three people, lived in their own houses (8), and belonged to economical classes B or C (11). Concerning the characteristics of the users, it was made evident that almost all were men (10), with ages characterizing them as young adults (average of 31.4 years old), low education level (average of 5.8 years of studies), single (7), and who did not exercise paid activities at the moment of the research (8). 

**Figure 1:**
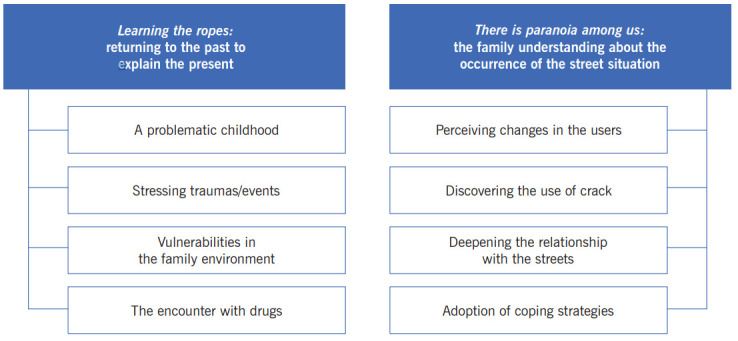
Categories and subcategories of the study

The analysis of the narratives showed that the family members understood the trajectory of their loved ones to the street situation from two perspectives which configure the study categories: *Learning the ropes: returning to the past to explain the present*, in which subjects narrated events of the childhood/adolescence of the users, conforming to an explicative model of the family about drug use, and *There is paranoia among us: the family understanding about the occurrence of the street situation*, in which the family members told specifically about the occurrence of the street situation from the start of crack use. The categories and subcategories generated may be visualized in Figure 1. 

### Learning the ropes: returning to the past to explain the present

When starting their family members' stories on the streets, the participants in this research presented contexts of suffering. These suffering contexts began in childhood and continued through the following cycles. The family members described several situations that defined "problematic childhoods". In this time, the primary complaints came from the schools and were generally related to the presence of behaviors considered inappropriate. The result of such problems and the interaction of the families with the schools culminated in processes of school changes going so far as school dropouts. The study participants also recognized the behavior of "staying on the streets" since childhood. The following excerpts seek to provide a panorama regarding accounts that compose this category: *Look, since a child he was always a rebel, he was always a problematic boy, you know? He did such things, so far-fetched [...]* (F8)*. He was always mischievous, he only did well in kindergarten. At this time, he was intelligent, dedicated... However, afterward, when he was bigger, he did not conclude his studies because he did not like studying and gave a lot of trouble at school. So he had to keep changing schools because the schools asked us to remove him [...] He went well in tests, but the teachers would tell me he would fail because of excessive absences* (F7)*.[...] he didn't want to study, was giving a lot of trouble, so I took him out of school (the user). However, since he was addicted to arcade games, he would take the shoeshine box and stay at the bus station, he would spend all day out of the house. He would say that the money he earned for shining shoes, he would spend playing* (F11*).*

The interviewees explained the “rebellious” behaviors as having been caused by traumas or stressor events. The interviewees' accounts describe situations of loss that adversely marked the life trajectories of the users, such as the death of close people and problems regarding fathers. *When his father died, he was eight years old. However, he was not at the wake or the burial, so he did not see his father in a coffin. However, their relationship was very strong; they had much love for each other, much admiration. Therefore, I think this may have harmed him a little* (F1). *When he started to rebel was when he found out that his father didn't want anything to do with him, didn't want to register him under his name, used to say he wasn't his, you know? Back then, I already had a son because I had become widowed. So I became very desperate, I did not want to have him, you know? I got angry at everything, I do not know if this passed over to him too* (F3). The childhood contexts were also described as contexts of "vulnerabilities in the family environment". The interviewees reported the presence of family conflicts, the absence of the fathers in the family environment, and about events that refer to the existence of incoherence in the establishment of boundaries, thus suggesting unstable family systems. The following accounts exemplify this context: *They never got along; I do not know, since childhood it seemed there was something wrong with him (the user) that he would keep insulted his father* (F3). *I always worked as a maid since my husband died, so I would stay a long time away from home, and he was alone much [...]* (EEL). *He was very much protected by his father, who didn't let no one hit him, always defended him when he did something that we didn't think was right [...]* (F1).

These childhoods in such vulnerable contexts have, as consequences, the "encounter with drugs". According to the accounts of the interviewees, since childhood the sick family members frequented places of greater risk; therefore the start of drug use took place in this context. We grouped two primary contents in this section: the influence of pairs in the initiation to drugs and the use of drugs by other family members. The following excerpts seek to give an example of the accounts grouped in this subcategory. *He always had these friendships, you know, these guys that use drugs, drink, are always at the bar door, he only had this type of friend. So I believe that they influenced a little* (F6). *Almost all her family members have had drug problems; her paternal grandfather died from drinking too much. Her aunt, around three months ago, was committed for drinking problems; of her five uncles, four drink. So I think drugs come with this sort of thing, they come from the family [...]. This is what I think was able to influence her* (F5).

### There is paranoia among us: the family understanding about the occurrence of the street situation

In this second moment of the family narratives, the interviewees listed events linked specifically to the occurrence of the street situation, from the initiation of crack use, as well as the coping mechanisms used by the families. Although aware of the drug use, the family member reported not knowing about the use of crack. Thus, a trajectory starts marked by a relationship in which, from the moment when it does not perceive that something is not well, the family moves to moments in which it perceives and recognizes this, as well as searches for solutions. We called this category "there is paranoia among us", because this process was described with much intensity where the family and the chemical dependent presented a confusing and ambivalent relationship, which, so far, has only intensified the problem. 

In the first subcategory of this moment, denominated "perceiving changes in the users", the family members recounted the first signs that a change was occurring in the behavior of the drug user family member: the practice of thefts of family belongings; aggression; physical changes such as lack of hygiene and weight loss; and also the situation of the user spending more time in street environments. The following accounts describe this moment: *He started to get more rebellious, rude with me, started with comebacks, speaking profanities, something he was not accustomed to doing. So he started to get rude and not to want to do anything that he used to like before* (EF1). *[...] he started to lose weight, lose his appetite, started to come home dirty, stinky, sometimes with a burn mark on his hands and mouth [...]* (EF6) *Then he started selling everything he had. I could not leave anything lying around, or he would sell it. So I started looking into it, and he was really messing with crack* (EF7). *So when he started using crack was when he started to spend more time on the streets, he started to not come home anymore. Then I thought he was using some very strong drug* (EF5).

Regarding the discovery of crack use, represented in the second subcategory of "discovering the use of crack", many family members said they did not recognize or know that the changes presented by the users were consequences of crack consumption. In some reports, these family members said they noticed the use of crack because of the environment, and most reported becoming aware because someone close to the family told them: *The only thing I noticed, because there was a room at the back of my house, and I would go there and see so much ash, so much ash, because the crack they use is with ash, right... I said: my God, what is this? [...] I would just work, went from home to work, from home to work, we did not even have time to watch TV, TV to know what a drug was. So I did not know at first that the change in him was because of crack* (EF3). *It was my brother-in-law, he gives lectures (former drug user who gives lectures for free in churches and schools about drug use prevention)... so he suspected, informed us of his suspicions, we went after him and caught him using drugs at school, right at the door of the school he attended* (F2). *I did not know. My luck was that my son-in-law said to me like this: look, I am going to tell you this, ma'am, and told me. When he told me, I went insane* (EF10).

In the third subcategory, "deepening the relationship with the streets", it stands out that no family member reported the occurrence of a "first episode" of the street situation but, to the contrary, they reported events that referred to the deepening of the relationship of the individuals with crack and the street situation, configured by periods of stability characterized by going to the streets and returning from the streets to the family environment: “*That's right, because before he would go to the streets, but always came back home, sometimes 1 AM, 2 AM, but always came back. Then, what happens? Before, he would come back 1 AM, 2 AM, but he started to stay two days, three days on the streets, you know. There were even times when he would go a month without showing up, without giving a sign of life, and, when he came back, he would come back in that situation, dirty, with his hands all hurt, and thin, really thin. And, when he arrived, he would fall into bed and sometimes sleep one or two days straight. Then he would get up, take a shower, and eat, but geez! He would eat whatever he saw in front of him, and then he would go off to the streets again*” (EF8). 

Regarding the "adoption of coping strategies", which represent the last subcategory, the family members narrated the ambivalence experienced in the face of the street situation episodes. They reported the adoption of different strategies in an individual, associated, or alternated manner with other family members, depending on the moment of the street situation in which the user was at - going to the street contexts or returning to the family environment. Thus, the following strategies were reported by family members: search or not on the streets, allow or not entry to the home, let go to the streets or not, and put them out on the streets or not. Describing a moment of ambivalence experienced by the family members regarding the strategies for searching or not on the streets: *"Oh, we searched for him, because we didn't sleep, at lunchtime, we remembered and wondered where he might be. What is he eating* (F4). *"So we searched for him, we would go out searching, telling many people 'if you see him, call me', I had a lot of help from people that saw him and came running to us, then I would call my husband, my husband was at work, he would leave work 'let's go because they say he's over there', so we went to get him* (EF45)". *I would wait for him, I would sleep, I still sleep to this day, I do not take medication, and I do not take anything. Because where he goes I cannot go, never did, I never went after him. So I ask God because where he goes, God goes in the right place* (EF10). 

## Discussion

Regarding the characteristics of the subjects interviewed, we verified that the majority of family members were mothers. This data is in accordance with results of a study carried out in Brazil which evaluated the relationship between cocaine and crack consumption with the dimensions of quality of life and social functioning of 1,560 young adults.(15) According to the study, the mothers seemed to be more "present" in the homes of crack users (74% of the cases) than on the homes of individuals in the general population, which leads to the belief that this family setting may be especially common in this population.(15) This data is important insofar as the strategies for preventing the street situation may be put into action by the health system in families that have some crack user as a member, viewing mothers as the primary figures to be considered in a family intervention process. On the other hand, this finding also indicates the possibility of parental overload, possibly leading to the occurrence of diseases and other aggravations to individual and family health. This was also pointed out by a study conducted in Canada about the impact of the use of alcohol and other drugs in the family dynamics, which found that most parents interviewed reported feelings of stress, anxiety, and depression, as well as other problems for the family as a whole.(16) 

Concerning the characteristics of the families, given the sample size, their profiles were similar both to the profile of the Brazilian population considering the results of the last National Survey by Sample of Households and to the profiles of families of crack users.(17,18) Regarding this last aspect, a qualitative study which analyzed the structures, relationships, and backgrounds of drug use in families of crack users through a genogram found very similar data, especially in regards to the economic classification and the low number of people in the first generation of the families that went to school.(18) The crack user profile found in the study was also consistent with the profile described in several studies about crack users in Brazil, including the predominance of men, the low education level, and the absence of formal work.(1,3,15,19)

This study allowed understanding that, in the perspective of family members, the trajectory of crack users to the street situation begins in childhood. Stories of traumatic events, behavior issues, and difficulty to remain in school since childhood explained the beginning of drug use and street living. 

The interpretation that people elaborate for a given experience of health-disease is the result of the different means through which they acquire their knowledge. Therefore, the explicative models are adopted by individuals with the perspective of offering an understanding of a given event, aiming the elaboration of the personal and social meaning of the experience of an aggravation.(20) For the family members that participated in this study, the events narrated about the childhood and adolescence of the crack user family member were perceived as moments of weakening that justify accepting and welcoming that child/youngster as the only way out. 

The vulnerability context of these families is also a relevant aspect that must be highlighted. A child's socioemotional development is related to their biological development and interactions with their environment since birth. Children who live in vulnerable environments are at greater risks of presenting difficulties in developing social, emotional, and self-perception competencies that aid them in the adoption of proper behaviors to the different contexts of society,(21)

In this context, when the behavior problems started to get in the way of school activities, the mother was called. However, the solutions found for these problems resulted in a constant changing of schools until school dropout. Elementary and high schools are closer to the families, so they may be in a strategic position to identify children and youngsters that need follow-up, contribute to the development of non-stigmatizing actions, and articulating with the community and health services to promote mental health and well-being to the young people.(22) 

School dropout favored the child or adolescent to intensify their time on the streets, thus justifying the beginning of drug use. In this initial period, the family recognized the drug use as a consequence of the environment frequented by the youngster. For being understood as a consequence of this troubled childhood, the drug use was not considered a problem at first. In a way, the family organized itself to be more tolerant in the sense of keeping the group united and meeting the needs of the group. 

In the perspective of the family systems, the psychosocial problems in the family are better understood and treated if analyzed from a circular perspective. In this perspective, each member of the family contributes with well- or ill-adapted interactions.(23) Following this logic, we understand that as the dependent family member intensified the drug use and their permanence in the streets, the family weakened their rules to keep the user in the family system. In other words, this means stating that the bad behaviors presented by the users may have acted as negative feedback, providing "information of the deviation", where the family system acted to "neutralize it" according to their belief system or way of understanding. This regulation or adjustment of the system, contained in Cybernetics, aims to maintain the survival of the family group, controlling the disturbances that afflict it, preventing changes from occurring beyond a threshold level that may change its organization.(24)

This way of dealing with the use of drugs allows understanding the second category of this study, characterized initially by difficulty in recognizing the use of crack and the intensification of the street situation. The difficulty in breaking with the functioning more tolerant with the use of drugs adopted by the family and the lack of information regarding crack determined the slowness to adopt more explicit coping measures. The lack of information about crack was one of the primary causes of family unawareness. The participants reported not having sufficient information to identify the alterations presented by the users. A study conducted with family members of crack users with the objective of analyzing the influence of the family environment on crack consumption found several family factors that contributed to the start of crack use.(25) Among them, the family disinformation and unawareness about drug use stood out, which prevented many families from acting in a sense to prevent/identify or even treat their family members, thus corroborating the data found in this study.(13) Therefore, the trajectory of the crack user to the streets is not understood by family members as a single moment; to the contrary, the streets have been present in the lives of the users since childhood. When it was no longer possible to maintain the family functioning more tolerant of drug use, the family needed to recognize the problem of intense crack use and street living, and adopt more restrictive measures. However, one may observe an ambivalence regarding such restrictive measures.

Often, families with psychosocial problems are helpless, tired, and inadequate in the face of their problems. Changing the way of functioning of the family so to seek other forms to handle the problem depends on their abilities to alter their perception of the problem. Systemic family nursing proposes that the nurse seek a position of collaboration with the family in the sense of seeking the necessary changes for coping with psychosocial problems. Under this perspective, the nurse that believes that the family unit can resolve their problems will not try to resolve them for the family because they know that the attempt to solve the family problems may inadvertently increase the family's sensation of helplessness and inadequacy, and promote dependency.(23)

This study, upon analyzing the family accounts about the trajectory of their loves ones involving the use of crack and the street situation found that the families adopt a meaning of explanation or belief that seems to function as adjustments to care for and keep the union and homeostasis of the family. The literature is clear when stating that the presence of drug use, violence, and ruptures in the family environment are good predictors for the occurrence of mental health problems for the entire family group.(5,19,16,18,25) Therefore, the participants in this study also reported some of these factors as causers of the behaviors of the crack users. However, this knowledge was an important element for family members to keep caring. The reduced number of participants in this study is a relevant limitation of the study, because it may be inferred that the family members are participating in little of the treatment of the crack user. And, in this sense, the analysis carried out is limited to family members who are still available to keep taking care of the sickened family member. 

## Conclusion.

The trajectory of the crack user to the street situation told from the narratives of family members of such users showed that the family adopts an explicative model for the drug use behavior and contact with the streets, based on the life story of this family member. The method adopted allowed exploring the family beliefs, their vulnerability context, and their efforts for keeping the family group united so to meet the needs of the entire group. The study reinforces the understanding of the family as a source of care, but also as a unit that required care to be able to exert its role in the recovery of crack users fully. Other studies are necessary to deepen this understanding and answer the questionings that arise upon verifying that, despite the evidence, families take long to recognize their loved ones' health problems.
